# Burden of Obesity in India: Need for Policy Changes to Attain Highest Possible Level of Health and Well‐Being

**DOI:** 10.1111/cob.70072

**Published:** 2026-02-02

**Authors:** Sanjay Kalra, Jothydev Kesavadev, Ramen Goel, Muffazal Lakdawala, Vinayak Agrawal, Neena Malhotra, Nitin Kapoor, Neeta Deshpande, Ankush Desai, Viswanathan Mohan, Rajesh Khadgawat, Balram Sharma

**Affiliations:** ^1^ Department of Endocrinology Bharti Hospital Karnal India; ^2^ Jothydev's Diabetes Research Centre Trivandrum India; ^3^ Center of Bariatric & Diabetes Surgery, Wockhardt Hospitals Mumbai India; ^4^ Sir H. N. Reliance Foundation Hospital Mumbai India; ^5^ Fortis Memorial Research Institute Gurugram India; ^6^ All India Institute of Medical Sciences New Delhi India; ^7^ Christian Medical College Vellore India; ^8^ Belgaum Diabetes Centre and CentraCare Institute of Diabetes, Obesity and Metabolic Health Belgaum India; ^9^ Department of Endocrinology Goa Medical College Bambolim India; ^10^ Madras Diabetes Research Foundation (ICMR Collaborating Centre of Excellence) and Dr. Mohan's Diabetes Specialities Centre (IDF Centre of Excellence in Diabetes Care) Chennai India; ^11^ Department of Endocrinology and Metabolism All India Institute of Medical Sciences New Delhi India; ^12^ SMS Medical College Jaipur India

**Keywords:** obesity in India, policy, white paper

## Abstract

The rise in obesity rates in India in the past 3–4 decades is alarming and obesity management needs transformation. A significant barrier is that obesity is often viewed in India as a lifestyle condition rather than a chronic disease, despite broad international recognition of this status.

We propose four overall recommendations to drive transformation of obesity management in India:

1. Change the narrative: Obesity is a complex, chronic disease, impacting multiple aspects of daily life to be addressed before complications or comorbidities arise
2. Move from a disease management to a health‐focused approach to reduce disparities in prevention and management of obesity
3. Invest in healthcare systems and the capacity to improve obesity management and prevention
4. Initiate, augment and scale the support given by the healthcare system to people living with obesity

Policy change is needed in India to ensure that obesity is adequately managed, to improve health of the overall population and reduce disease burden and healthcare costs. Standardised treatment guidelines and algorithms will ensure that individuals with obesity receive up‐to‐date and consistent support. The burden of obesity can only be addressed through acceptance of obesity as a disease and collaboration across healthcare and policy stakeholders, with significant resource commitments at every level.

**Abbreviations**
BMI: body mass index
CVD: cardiovascular disease
GDP: gross domestic product
HCP: healthcare professional
ICMR‐INDIAB: Indian Council of Medical Research–India Diabetes
NCDs: non‐communicable diseases
NP‐NCD: National Programme for Prevention and Control of Non‐Communicable Diseases
PCP: primary care physician
T2D: type 2 diabetes
WC: waist circumference
WHO: World Health Organization
WOF: World Obesity Federation

## 1 Introduction

### 1.1 The Global Burden of Obesity

Obesity was recognised as a disease in 1948 by the World Health Organization (WHO) and by the American Medical Association in 2013 [1, 2]. The WHO defines obesity as a chronic, complex disease of excessive fat deposits, presenting a health risk. It is progressive and relapsing and associated with an increased risk of non‐communicable diseases (NCDs), including type 2 diabetes (T2D) and cardiovascular disease (CVD) [3, 4, 5, 6]. In Asian populations, individuals with a body mass index (BMI) of ≥ 25 kg/m^2^ are considered to be living with obesity, with those with a BMI of 23–24.9 kg/m^2^ categorised as overweight (WHO cut‐offs for obesity and overweight, based largely on data from Caucasian populations, are ≥ 30 and ≥ 25 kg/m^2^, respectively) [3, 5, 7, 8, 9].

The World Obesity Federation (WOF) estimates that by 2030, 1.1 billion adults will be living with obesity worldwide [6]. By 2060, the global cost of overweight and obesity is predicted to be over ₹1544 trillion (converted on 2/1/2025 at 1 United States dollar to 85.79 Indian rupees; rate used throughout) [10].

Altogether, given the chronic, progressive nature of obesity, which poses both clinical and economic burden, early recognition and diagnosis are crucial to improve outcomes. Proactive screening for high BMI or central adiposity can facilitate timely intervention before severe comorbidities develop [11, 12]. For instance, clinical guidelines recommend routine obesity screening in adults and referral for appropriate management [11].

### 1.2 The Burden of Obesity in India

#### 1.2.1 Prevalence of Obesity in India

Obesity‐related comorbidities have been demonstrated at lower BMI in India compared with Western countries and as such established consensus has been to define obesity in India as BMI ≥ 25 kg/m^2^ (with overweight categorised as BMI 23–24.9 kg/m^2^) [7, 8, 9]. Using that definition, the Indian Council of Medical Research–India Diabetes (ICMR‐INDIAB) study estimated that in 2021 there were 254 million individuals with generalised obesity (BMI ≥ 25 kg/m^2^) and 351 million with abdominal obesity, defined as waist circumference (WC) ≥ 90 cm in males and ≥ 80 cm in females [13].

Even using the global threshold of ≥ 30 kg/m^2^, India had the third largest total number of adults living with obesity worldwide in 2022 and the rise in obesity rates is cause for concern, increasing from 1.2% to 9.8% in women and from 0.5% to 5.4% in men between 1990 and 2022 [6, 14, 15, 16]. Obesity rates are predicted to rise substantially and by 2050 an estimated 17.4% of women and 12.1% of men are estimated to be living with obesity in India [17].

The future burden is particularly concerning, with prevalence in children and adolescents (aged 5–19 years) increasing from 0.1% to 3.1% in girls and from 0.2% to 3.7% in boys between 1990 and 2022, to a total of 12.5 million children and adolescents with obesity [14, 16, 18, 19, 20]. Rates of obesity are also higher in urban areas than in rural areas [13, 15, 18, 19, 21].

However, the true burden of obesity in India may be under‐represented. Following a recent Delphi consultation, the new consensus is to categorise obesity into two stages. Stage 1 is defined by increased adiposity and BMI ≥ 23 kg/m^2^ without any appreciable impact on daily activity or organ function. Stage 2 also uses a BMI cut‐off of ≥ 23 kg/m^2^, but in addition requires an excess WC measurement or waist‐to‐height ratio and either the presence of ≥ 1 symptom indicative of an impact on daily activity (shortness of breath, palpitations, or musculoskeletal symptoms) or the presence of an obesity‐related comorbidity (e.g., T2D or hypertension), thus integrating clinical considerations along with anthropometric measurements [22]. The BMI cut‐off used in these stages would more accurately represent the burden of obesity in India.

Further complicating the picture is the ‘thin‐fat’ obesity phenotype in India, defined by a normal BMI but with increased and unhealthy distribution of body fat, higher visceral adipose tissue and similar cardiometabolic risk as for typical obesity. Prevalence is approximately 32%, requiring specific diagnosis but similar management to typical obesity [23, 24].

#### 1.2.2 Morbidity and Mortality Associated With Obesity in India

In 2019, there were 579 074 adult deaths in India from NCDs attributed to overweight or obesity, including relating to diabetes, CVD, stroke and cancer. The related person‐years lost (disability‐adjusted life‐years) was 21 005 319 in the same year [18]. Obesity is ranked in the top 5 risk factors for premature death due to T2D and to cancer (and in the top 15 for ischaemic heart disease and strokes) [6]. In terms of cancer specifically, obesity is a risk factor for colorectal, endometrial, gallbladder, liver, oesophageal, pancreatic and post‐menopausal breast cancer [25].

There are also specific risks associated with obesity in certain populations: pregnant women with obesity, for example, are at risk of conditions such as gestational diabetes and hypertensive disorders of pregnancy and their foetus is at risk of stillbirth or infant death, or congenital abnormalities [26].

#### 1.2.3 The Economic Burden of Obesity in India

The cost of overweight and obesity in India was estimated at ₹2.5 trillion in 2019, including ₹199 billion of direct medical/non‐medical costs and ₹2.3 trillion of indirect costs (relating to absenteeism, presenteeism and premature mortality), representing 1.02% of the gross domestic product (GDP) [27]. By 2060, costs are predicted to rise to ₹72 trillion (₹3.7 trillion direct and ₹68 trillion indirect, the latter largely premature mortality), representing a 29‐fold increase and 2.47% of India's GDP [27].

#### 1.2.4 The Challenge of Obesity in India

In India, obesity is often viewed as a lifestyle condition rather than a disease by the general public, policymakers and key healthcare stakeholders, with no need for healthcare professional (HCP) intervention, despite broad international recognition of its disease status [3, 4, 28, 29]. Obesity and weight loss are often seen by people living with obesity in India as a personal failing for which they are wholly responsible for addressing—it is associated with social discrimination and humiliation, acting as a barrier to seeking assistance from HCPs and is complicated by socio‐economic factors relating to long‐standing poverty in the country [29, 30, 31]. Furthermore, in some South Asian communities, excess weight can be perceived as a sign of prosperity rather than a medical concern [32]. Additionally, many HCPs do not consider obesity as a disease, which impedes care, including the initiation of appropriate treatment [33]. While it is true that unhealthy diet and physical inactivity contribute to the obesity epidemic, the etiological factors involved are more diverse than simple lifestyle‐related factors like overeating or lack of exercise [34, 35, 36].

Several national bodies in India have published a consensus for recognising obesity as a chronic disease, highlighting the challenges in managing obesity until recognition is gained and providing a call to action for obesity prevention and management, which we echo here. These organisations include the Endocrine Society of India [26] and regional organisations such as the South Asian Federation of Endocrine Societies (with the Kathmandu declaration) [37] and the South Asian Obesity Forum (with the Colombo declaration) [38, 39]. The latter highlighted the need for an obesity strategy that is partnership‐based, person‐centred, public‐inclusive and policy‐concordant and included consideration of the psychological and psychosocial aspects of obesity management [39].

In 2025, Indian Prime Minister Narendra Modi noted obesity as a national issue in his public ‘Mann Ki Baat’ address, stating ‘By making small changes in our food habits, we can make our future stronger, fitter and disease‐free’. This was coupled with a public health statement highlighting national schemes relating to health and obesity awareness and management, including the National Programme for Prevention and Control of Non‐Communicable Diseases (NP‐NCD), the Ministry of Ayush promotion of traditional and holistic health practices, the POSHAN Abhiyaan initiative for preventing childhood obesity, the Ministry of Youth Affairs and Sports focus on physical fitness and the Food Safety and Standards Authority of India regulation of food for public health [40].

However, despite these important national calls to action, there remains a lack of consistent guidelines for obesity management or definition in India, with consensus groups proposing different staging systems or management algorithms (e.g., Deshpande et al. propose using the Edmonton Obesity Staging System based on functional limitations and the presence of obesity‐related comorbidities to supplement anthropometric measurements) [30]. Although obesity training for HCPs was introduced in 2018, the application of the training is inconsistent and not yet felt in clinical practice [41]. As a result, specific obesity management settings are lacking. Regional disparities also mean access is not consistent or equal [42, 43, 44].

#### 1.2.5 Addressing the Burden of Obesity in India

Given the growing social, economic and health burdens associated with obesity, it is essential to ensure that people living with it receive appropriate management. To address the obesity epidemic in India, transformative and comprehensive policy changes, along with greater awareness, are needed from the government and the general population. This must begin with recognising obesity as a disease in India and incorporating it into the Government of India's NP‐NCD. Standardised guidelines will be key to driving change in obesity, especially given the multiple stakeholders involved. Prevention and management should be data‐driven and evidence‐based; there is a need for more alignment from the research community regarding data collection on the societal costs of obesity.

To address the burden of obesity in the Asia–Pacific region, in 2022, Novo Nordisk conducted the Obesity Disease Burden study in collaboration with a committee of regional experts. The study included a survey of adults living with overweight and obesity (see Supporting Information appendix for methodology, survey questions and results) and a modelling analysis (published by Yoong et al. [45]).

An Obesity Advocacy Group forum of local experts, including endocrinologists, bariatric surgeons, cardiologists, diabetologists, regional practitioners and representatives from the Indian Council of Medical Research and the All India Association for Advancing Research in Obesity, was convened to discuss the India‐specific findings and the gaps in obesity management and to put forward a series of recommendations pertaining to the prevention, treatment and management of obesity in India.

## 2 Methods

The policy recommendations presented below were formulated through expert discussion in the Obesity Advocacy Group forum, organised by Novo Nordisk on September 28th, 2024, in New Delhi. The author group for this white paper represents the attendees of that forum, following consensus discussion on the recommendations and their priority. Supporting evidence was obtained via literature review and from the Obesity Disease Burden study (using India‐specific data from the unpublished survey section of the study and the previously published modelling analysis) [45].

## 3 Policy Recommendations for Transforming Obesity Management in India

Four overall recommendations with associated goals have been identified to drive the transformation of obesity management in India:

1. Change the narrative: Obesity is a complex, chronic disease impacting multiple aspects of daily life to be addressed before complications or comorbidities arise
2. Move from a disease management to a health‐focused approach to reduce disparities in prevention and management of obesity
3. Invest in healthcare systems and the capacity to improve obesity management and prevention.
4. Initiate, augment and scale the support given by the healthcare system to people living with obesity

**FIGURE 1:**
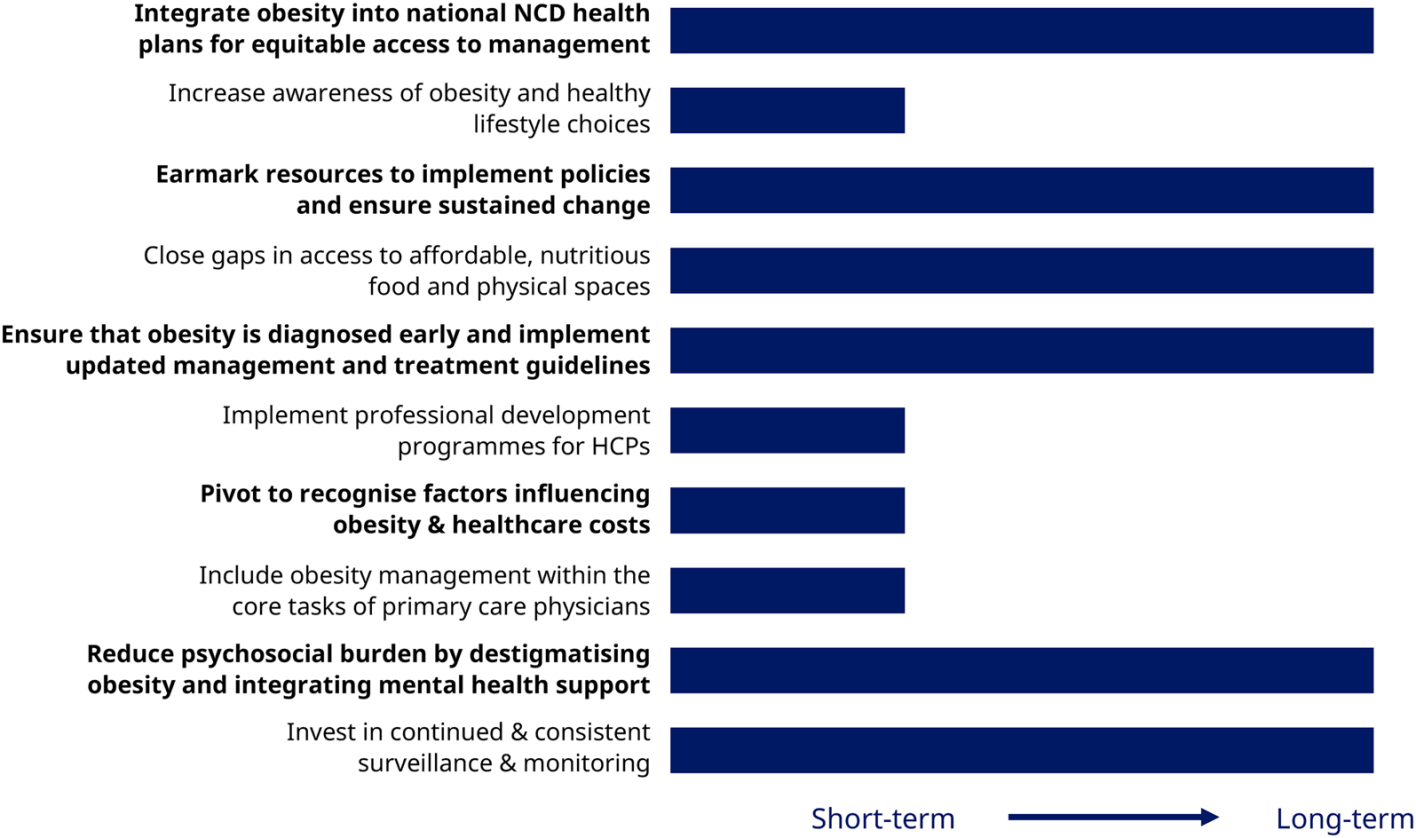
Ten interventions in obesity care in India. HCP, healthcare professional; NCD, non‐communicable disease.

Within these recommendations, 10 specific interventions have been chosen, on which efforts must be focused. Each has been assigned to one of the following levels that illustrate scale: ‘Mega’, ‘Macro’, ‘Meso’ and ‘Micro’. Each has also been designated as having either short‐ or long‐term implementation and impact, summarised in Figure 1.

### 3.1 Change the Narrative: Obesity Is a Complex, Chronic Disease, Impacting Multiple Aspects of Daily Life To Be Addressed Before Complications or Comorbidities Arise

#### 3.1.1 Develop Obesity‐Specific National Health Plans That Ensure Equitable Access to Management, and Integrate Obesity Within National NCD Plans (Macro‐Level; Long‐Term)

**FIGURE 2:**
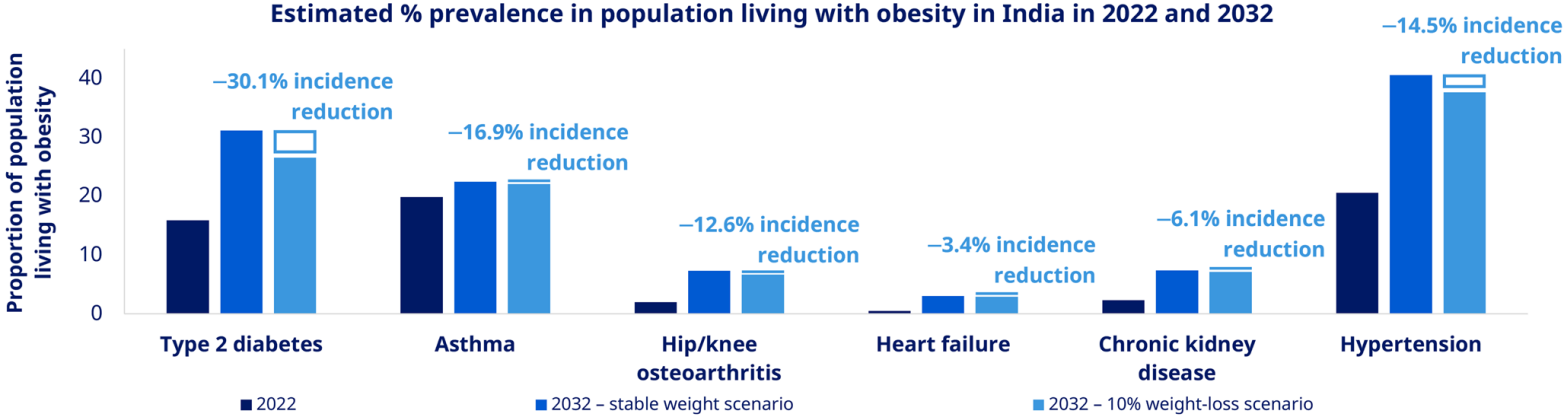
Effect of 10% weight loss on 10‐year incidence. Based on data published in Yoong et al. [45].

Incidence of key NCDs including T2D, hypertension and heart failure is projected to double by 2032 [45]. For example, it was estimated that there were 35.4 million cases of T2D in 2022. With no intervention, 69.3 million cases are predicted by 2032. However, if a population‐level 10% weight loss is achieved (as proposed in the model), cases will instead rise to 59.1 million, a 30.1% incidence reduction (Figure 2).

The same analysis estimated that direct medical costs for obesity‐related comorbidities in 2022 were ₹2.0 trillion, projected to increase to ₹3.8 trillion in 2032 (from ₹8923 to ₹17 759 per person living with obesity per year). With a 10% weight loss, costs are predicted to rise to only ₹3.5 trillion in 2032—still a high burden but saving ₹257 billion and cumulatively saving ₹1.5 trillion from 2022 to 2032 (Table 1) [45]. Savings were predicted to be highest in the T2D and hypertension groups, at ₹94 billion and ₹98 billion, respectively, in 2032 alone [45].

**TABLE 1:** Direct medical costs in 2022 and in 2032.

Population living with obesity	2022		2032: No intervention		2032: 10% weight loss		Cost savings: 10% weight loss	
	Direct medical costs (INR)	Cost per person (INR)	Direct medical costs (INR)	Cost per person (INR)	Direct medical costs (INR)	Cost per person (INR)	In 2032	Cumulative over 10 years
223 million	2.0 trillion	8922	3.8 trillion	17 759	3.5 trillion	16 472	257 billion	1.5 trillion

*Note:* Adapted from Yoong et al. [45]. Original publication reported costs in USD. Conversion to INR at 1 USD = 85.79 INR (conversion date 02/01/2025).

Abbreviations: INR, Indian rupee; USD, United States dollars.

To reduce the anticipated impact resulting from the burden of obesity and the substantial associated healthcare costs, the Indian government needs to further enhance national health plans, develop policies to recognise obesity as a disease and integrate obesity management into prevention‐related actions within national NCD plans. Obesity is currently included in the Indian NP‐NCD as a risk factor for other NCDs. These plans must instead be updated to consider obesity itself as a disease and to manage it from a broader focus on health and its link to other NCDs.

#### 3.1.2 Pivot Governmental and Societal Views on Obesity to Recognise the Factors That Can Influence Obesity and Related Healthcare Costs (Mega‐Level; Short‐Term)

Obesity needs to be recognised as a disease in India to ensure that it receives due attention on a governmental and societal level.

In India, perception remains that obesity is a lifestyle condition, but the perspective on this needs to change to remove stigma and better support individuals (see next intervention) and to reduce the overall burden of obesity both on their health and on healthcare costs. Even after overcoming the impact of stigma and discrimination, sustaining weight loss is a psychological and physiological challenge beyond achieving the initial loss due to environmental, social pressures and genetic predisposition. Of the 403 survey respondents for the aforementioned Obesity Disease Burden study, 84.0% attempted weight loss using diet, exercise, or medical treatment, but only 4.7% had maintained their weight loss of 5%–15% for at least a year (Table S1). To support individuals with their weight loss, education around the impact of obesity as a disease and the most effective ways to manage it is needed.

This paradigm shift, which is complicated by disparities in accessibility and inequalities in healthcare use, health‐seeking behaviours and healthcare expenditures between rural and urban settings and between income levels, needs to cascade from the top‐down. Structural changes on a governmental and healthcare system level and changes to HCP education are needed to address these and the impacts of obesity on healthcare costs.

Obesity management also needs to be expanded beyond the prism of healthcare only and integrated into other aspects of life, with education, community and governmental support to actualise and maintain real reductions in obesity rates at home and to translate into reduced costs and burdens of obesity‐related diseases.

Individuals, employers, businesses, HCPs, nutritionists, surgeons, researchers, the pharmaceutical industry and policymakers should collaborate to address weight management and to create environments that will improve obesity treatment.

#### 3.1.3 Reduce Psychosocial Burden by Destigmatising Obesity and Integrating Mental Health Support to Improve Management Pathways (Micro‐Level; Long‐Term)

**FIGURE 3:**
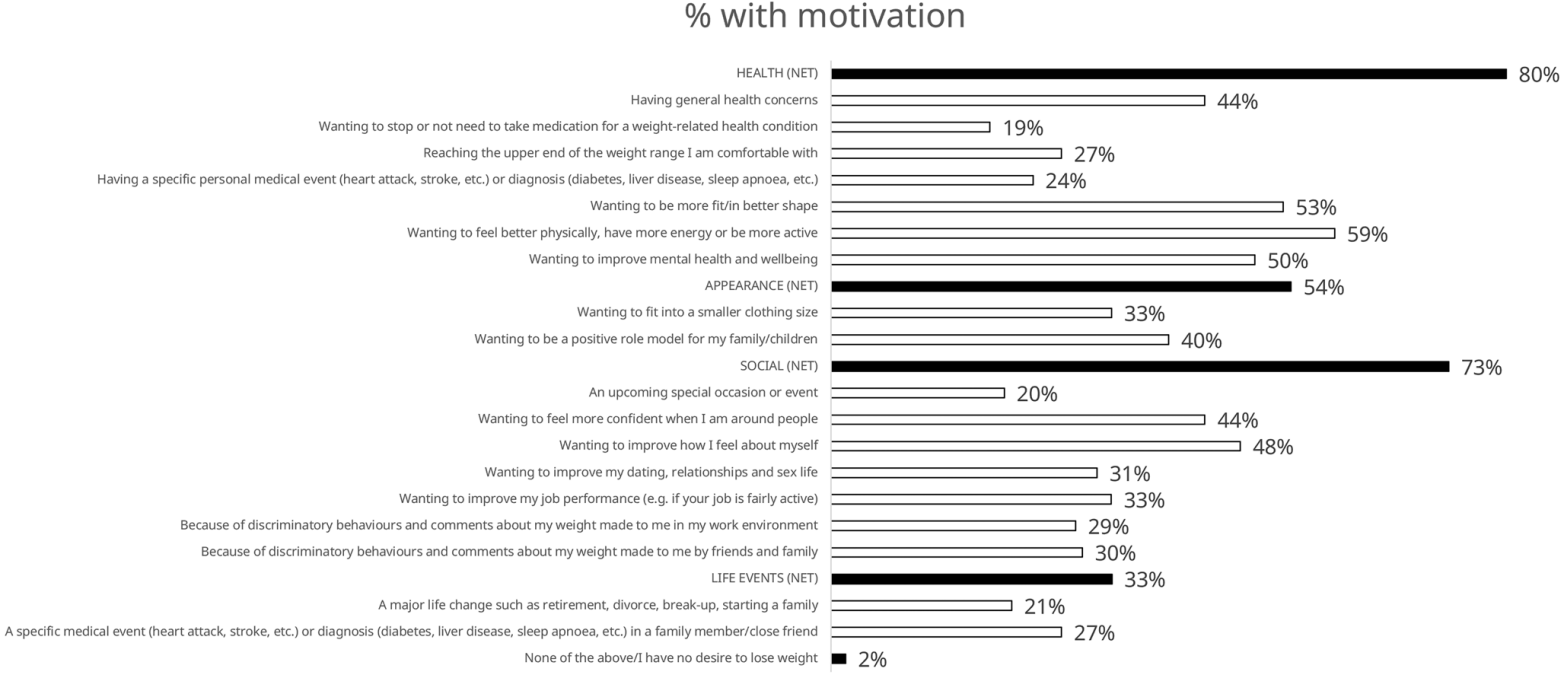
Responses to the survey question ‘Which of the following, if any, have motivated you the most to make a plan to lose weight, today or in the past?’ in respondents who were currently trying to lose weight.

Although health reasons were commonly reported as motivators for weight loss, survey respondents trying to lose weight were also often motivated by social reasons, with 28.9% responding that they were motivated by discriminatory behaviours/comments about their weight in the work environment and 29.7% by discriminatory behaviours/comments from friends and family (Figure 3; Table S1).

The stigma of obesity may mean that people are reluctant to proactively discuss their weight with their HCP or with their support network. Population‐level anti‐stigma initiatives are needed to reframe how obesity/overweight is perceived in India and move away from the perception that it is a lifestyle choice and solely an individual's responsibility, to address cases of discrimination as the motivator for weight loss and to offer mental health support for people living with obesity. This stigma needs to be addressed on a societal level but also needs to be addressed with HCPs through training to ensure that they listen to patients and offer advice and management of obesity without discrimination or promoting a feeling of shame with the individual.

In addition, the social stigma aspect of obesity and associated psychosocial burden, coupled with the psychological challenges of weight loss (which can lead to mood and motivational disorders, low self‐esteem or body image issues and eating disorders) [29], requires involvement of mental health professionals in the pathway of obesity management, with psychologists and counsellors utilised as part of the support network for individuals with obesity.

### 3.2 Move From a Disease Management to Health‐Focused Approach to Reduce Disparities in Prevention and Management of Obesity

#### 3.2.1 Earmark Resources to Implement Policies and Ensure Sustained Change (Macro‐Level; Long‐Term)

Sufficient financial and human resources need to be allocated to support successful implementation of these policies in the long term. Implementation of obesity‐related policies is key for integration into the Indian healthcare system and requires public funding and governmental strategic input.

#### 3.2.2 Close Gaps in Access to Affordable, Nutritious Food and Spaces for Physical Activity (Macro‐Level; Long‐Term)

Many aspects within urban environments are determinants of health and adopting a public health approach and investing in urban health development can reduce individual vulnerabilities to obesogenic environments.

Access to essential infrastructure and services is needed for all individuals and the development of social mechanisms and sustainable models in the urban health setting can help to treat and prevent obesity. Healthy diets should be affordable and accessible to all socioeconomic groups; nutritious foods should be subsidised and local production should be encouraged to reduce costs and improve availability [46, 47].

Public access to and availability of safe spaces for physical activity should be increased as well, supported with group education and group‐based physical activity, which can be effective in increasing physical activity among disadvantaged groups. This should be made readily available across the country.

### 3.3 Invest in Healthcare Systems and Capacity to Improve Obesity Management and Prevention

#### 3.3.1 Implement Professional Development Programmes for HCPs (Meso‐Level; Short‐Term)

In total, 34.5% of survey respondents reported that they had not spoken to an HCP about their weight in the past 5 years. Similarly, 35.1% of respondents had not received an obesity diagnosis from a medical doctor or qualified HCP.

Continuous professional development is key for HCPs, to ensure that they are aligned with the latest treatment paradigms for obesity management. This includes training to manage difficult conversations related to weight and obesity with their patients and to help patients focus on both short‐ and longer‐term health goals. Training should be combined with the latest changes to clinical guidelines and the expansion of the role of HCPs in managing obesity and both integrated into the curriculum for training new HCPs and into continued education for established HCPs.

#### 3.3.2 Invest in Continued and Consistent Surveillance and Monitoring to Strengthen Understanding and Drive Research (Mega‐Level; Long‐Term)

Population‐based obesity surveillance systems that are rapid, robust and secure are critical for monitoring trends in obesity management and in determining the overall effect of obesity prevention strategies. Additional research is also needed to prevent or manage the ‘thin‐fat’ obesity phenotype and should be conducted in conjunction with academic and international institutions. Funding is needed to further obesity research as a whole in India, from both a national level and international organisations such as the WHO, UNICEF and the WOF.

### 3.4 Initiate, Augment and Scale the Support Given by the Healthcare System to People Living With Obesity

#### 3.4.1 Increase Awareness of Obesity and Healthy Lifestyle Choices (Mega‐Level; Short‐Term)

Dissemination of information on obesity, health services and health‐promoting behaviour needs to be improved in India and should not purely be based within the healthcare system, but with community and government support. Education on obesity and its related complications should be improved across the whole spectrum of daily life, translating to healthy choices and lifestyle at home. This needs to begin at a young age—childhood obesity rates are rising and households are often not aware of what defines obesity, both in adults and children. Healthy eating habits should be encouraged from school age, with nutrition education for children and parents, increasing physical activity programmes, curation of school meals that meet nutritional standards and education on obesity and its association with obesity‐related diseases included in school curriculums.

Information can be disseminated digitally, with specific obesity educators or health kiosks in hospitals, schools, airports, etc. where individuals can gain information easily. Food labels should have clear nutritional information to improve public awareness on diet and health. Further education should be made available to help individuals and their families make more informed choices and highlight the benefits of a healthy lifestyle beyond weight loss.

Comprehensive public health education campaigns on obesity through mass media and community outreach should be used to motivate individuals to seek health information and services, thereby promoting healthy lifestyles. This information needs to be informed and framed by medically sound and reviewed advice, avoiding potentially spurious or harmful weight‐loss solutions.

Targeted campaigns aimed at specific populations, such as menopausal women or women who are pregnant or postpartum, would be invaluable for educating groups and developing support networks. An awareness campaign is also needed for ‘thin‐fat’ obesity, defined as those who may not be classified as having obesity by measuring body weight or BMI but still have excess body fat, to increase public knowledge of this phenotype, the health complications associated with it and management strategies.

However, it has been established that diet and exercise alone are insufficient for successful and sustained weight loss [48, 49]. Genetic correlates of obesity also impact weight, as does environment and improved dissemination of information should include these aspects of obesity and weight loss. Survey data demonstrated that dietary (76.3%) and exercise (80.6%) interventions are more commonly attempted than medical interventions (50.9%; e.g., prescription/over‐the‐counter medications, behavioural therapy/psychotherapy, or surgery) for weight loss and respondents considered these to be the most effective options, despite evidence to the contrary (Table S1) and it was demonstrated in the ACTION APAC study that HCPs recommended improved eating habits and increased physical activity as the top weight management methods and they and people with obesity preferred these routes over medications [31]. Physical activity/exercise may also be difficult to manage for some individuals with obesity, due to either the severity of their obesity or other conditions that limit physical activity. Healthcare experts in the obesity field in India need to lead the dialogue on obesity and be closely involved in the development of public health initiatives to ensure that they are driven by health and science.

To assist with shifting the perception of obesity and increased uptake of obesity treatments, health insurance policies should be expanded to include obesity treatments (medical interventions; surgical treatment for weight loss has been covered since 2020) [43]. This coverage should empower and encourage individuals with obesity to seek help from HCPs.

#### 3.4.2 Ensure That Obesity Is Diagnosed Early and Implement Updated Management and Treatment Guidelines (Meso‐Level; Long‐Term)

Diagnosis of obesity is reliant on people living with the disease engaging with their primary care physician (PCP) to discuss their weight and assessment primarily through BMI and other anthropometric measurements. Increasing awareness of obesity and its related complications, as well as reducing social stigma related to weight, is needed to improve the rate of people comfortable coming forward to discuss with their HCPs.

However, to improve early diagnosis and management, HCPs need to ensure they are discussing weight with their patients, listening to patient concerns and implementing the best tools at their disposal.

Formalised and consistent treatment guidelines, HCP engagement and follow‐up are needed to implement and improve obesity management in India. A trained workforce of obesity educators and dedicated obesity clinics are highly recommended. To assist with accurate and early diagnosis, clinical measures (such as presence of comorbidities, functional impacts) should be included in diagnostic criteria in tandem with anthropometric measures, as part of the HCP toolkit. In managing obesity, a broader range of obesity‐related health outcomes should be assessed for individuals rather than just specific weight‐loss goals (e.g., glycaemic profile, lipid profile, or blood pressure outcomes). This requires consistent detection and treatment, including weight measurement and screening, nutrition guidance, physical activity, psychological support, counselling, pharmacotherapy and surgery, as needed and as appropriate to local practice and populations. Therefore, the approach, both to diagnosis and management, while starting with an individual's PCP, needs to be multidisciplinary.

Guidance should extend to childhood obesity and to pregnancy and postpartum weight gain, as and when relevant. The future burden of obesity as a result of rising rates of childhood obesity, or associated with obesity in pregnancy, the increased risk to mother and child associated with obesity during pregnancy and weight gain postpartum are not well supported. Management and treatment guidelines should therefore also incorporate the advice of paediatricians and obstetricians to ensure these elements are included in management algorithms.

Increasing healthcare budgets and resources at the hospital level will be necessary to ensure the availability of adjunct interventions. Expanding the network of HCPs who can prescribe adjunct interventions will also be key to supporting PCPs and secondary specialists in delivering effective support for people living with obesity.

#### 3.4.3 Include Obesity Management Within the Core Tasks of Primary Care Physicians (Micro‐Level; Short‐Term)

PCPs are one of the main HCP groups with whom people in India discuss their weight (55.1% of survey respondents who have discussed their weight with an HCP did so with their PCP; Table S1).

The World Obesity Federation recommends that obesity management be included among the main tasks for PCPs to support the prevention and management of obesity. This should be achieved both through providing PCPs with the tools they need to take anthropometric measurements (i.e., equipment for taking height and weight, WC measurements) and training them to investigate more clinical determinants of obesity (i.e., questioning physical impairment, investigating obesity‐related comorbidities). These would incorporate obesity management into consultations when relevant, either when triggered by patients inquiring about their weight and obesity or through their own questions and investigations. By acting early and proactively, PCPs may be able to help either to avoid obesity and obesity‐related complications or commence treatment to manage them. This is particularly important in rural areas with limited access to specialists; sustained monitoring and management of obesity at all levels, in both public and private healthcare, will help to avoid undue burden on the healthcare system, both in terms of costs and capacity.

Expansion of treatment guidelines and implementation of consistent algorithms (e.g., the algorithm proposed by Deshpande et al. [30]) developed in collaboration with HCPs, which start with primary care, will be of particular importance in addressing this goal. Considering the ubiquitous nature of obesity, standardised and algorithmic management of obesity should be compulsory. This will ensure that all stakeholders involved are aligned on how to manage obesity.

Resource allocation to build these capacity and capability initiatives should be prioritised in a sustainable manner. These recommendations and interventions require a broad range of stakeholders, including endocrinologists, physicians, nutritionists, bariatric surgeons, diabetologists, gynaecologists, nurses, psychologists and other specialities, for them to be effective; it is not just the responsibility of one speciality alone. Unity is needed, with policy action at the national and clinic level and collaboration between HCPs at all levels—from primary care to focusing in on tertiary care at the local level. Physicians across various specialities should be proficient in identifying obesity as a disease to either develop a collaborative and comprehensive treatment strategy or refer the patient to an obesity specialist.

It will be important to establish clear metrics to track and assess the implementation of these recommendations. Adjustments may be necessary to calibrate these progressively and periodically. Engagement at a community and local level will be invaluable when designing and implementing obesity management and prevention programmes to make sure that they are applicable to specific community needs.

## 4 Conclusions

The Obesity Disease Burden study underlines the clear need for healthcare reform and corresponding resource commitments in India to make population‐wide improvements in health outcomes achievable in people living with obesity and to reduce the burden of obesity. The consequences of insufficient action will be substantial and costly, from both a public health and economic perspective.

### 4.1 Top‐Down Obesity Strategy

The WHO, along with the USA, Canada, Japan and most European countries, recognises that obesity is a disease and has developed or is developing frameworks to address the burden of obesity. India needs to follow their example and recognising obesity as a chronic, complex disease is the first step for it to be incorporated into India's NP‐NCD plans. Focusing on obesity and reducing the national burden could also help India in addressing the United Nations Sustainable Development Goals, specifically Goal 3 ‘Ensure healthy lives and promote well‐being for all at all ages’ [46].

### 4.2 Education and Public Awareness

The belief that diet and exercise are sufficient to successfully achieve and maintain weight loss and the societal pressures and discrimination associated with obesity are significant barriers for progress. To move forward and take control of the scale of the obesity issue in India, education and public health campaigns will be key to ensuring that everyone understands the burden and complexity of obesity.

### 4.3 Healthcare Reform

Transformative policy change is needed in India to ensure that obesity is adequately managed, to improve overall population health and to reduce economic burden and healthcare use. The NP‐NCD should focus on obesity as a disease as with other NCDs, rather than simply a condition that is a risk factor for other NCDs. Standardised treatment guidelines and algorithms will ensure that individuals with obesity receive the most up‐to‐date and consistent support. Ultimately, the burden of obesity can only be addressed through collaboration across the gamut of healthcare and policy stakeholders, with significant resource commitments at every level. It is achievable and the time to act is now.

## Funding

This work was supported by the Novo Nordisk.

## Conflicts of Interest

S.K. has received speaker fees from Eli Lilly and Novo Nordisk. J.K. has received honorarium from Abbott Diabetes Care, Eli Lilly, Medtronic, Novo Nordisk, Roche and Sanofi. R.G. has received honorarium from Eli Lilly, Ethicon Endosurgery and Novo Nordisk, consulting fees from Eli Lilly, support for attending meetings and/or travel from Novo Nordisk, has participated in data safety monitoring or advisory board meetings for Ethicon Endosurgery and Novo Nordisk and participated in clinical trials for Eli Lilly and Novo Nordisk. M.L. has received honorarium from Novo Nordisk, Medtronic, Ethicon, Johnson & Johnson and Meril and participated in clinical trials for Novo Nordisk. V.A., N.M., A.D. and R.K. report no conflicts of interest. N.K. has received research grants from the Global Alliance for Chronic diseases, the Indian Council of Medical Research and the National Health and Medical Research Council of Australia and has been a principal investigator for Amgen, Eli Lilly, Novartis and Novo Nordisk. N.D. has received honorarium from Eli Lilly and Novo Nordisk. V.M. has acted as consultant and speaker, received research or educational grants from Abbott, Alembic, Apex, Bayer, Biocon, Boehringer Ingelheim, Cipla, Dr. Reddy's, Eli Lilly, Emcure, Fourrts, Franco Indian, GlaxoSmithKline, Glenmark, INTAS, IPCA, Lifescan, Lupin, Mankind, Medtronics, MSD, Novartis, Novo Nordisk, Primus, Roche, Sanofi, Servier, Sun Pharma, Torrent, USV, Wockhartd and Zydus. B.S. has received research grants or contracts, consulting fees and honorarium from Novo Nordisk.

## Supporting information


**Data S1:** Supporting Information.

## Data Availability

Data sharing not applicable to this article as no datasets were generated or analysed during the current study.

## References

[1] American Medical Association , Recognition of Obesity as a Disease H‐440.842 (American Medical Association, 2025).

[2] W. P. James , “WHO Recognition of the Global Obesity Epidemic,” International Journal of Obesity (London, England: 2005) 32, no. Suppl 7 (2008): S120–S126, 10.1038/ijo.2008.247. 19136980

[3] World Health Organization , Obesity and Overweight (World Health Organization, 2024), https://www.who.int/news‐room/fact‐sheets/detail/obesity‐and‐overweight.

[4] G. A. Bray , K. K. Kim , and J. P. H. Wilding , “Obesity: A Chronic Relapsing Progressive Disease Process. A Position Statement of the World Obesity Federation,” Obesity Reviews 18, no. 7 (2017): 715–723, 10.1111/obr.12551. 28489290

[5] Prospective Studies Collaboration , “Body‐Mass Index and Cause‐Specific Mortality in 900,000 Adults: Collaborative Analyses of 57 Prospective Studies,” Lancet 373, no. 9669 (2009): 1083–1096, 10.1016/S0140-6736(09)60318-4. 19299006 PMC2662372

[6] World Obesity Federation , World Obesity Atlas 2025 (World Obesity Federation, 2025), https://data.worldobesity.org/publications/world‐obesity‐atlas‐2025‐v6.pdf.

[7] A. Misra , P. Chowbey , B. M. Makkar , et al., “Consensus Statement for Diagnosis of Obesity, Abdominal Obesity and the Metabolic Syndrome for Asian Indians and Recommendations for Physical Activity, Medical and Surgical Management,” Journal of the Association of Physicians of India 57 (2009): 163–170. 19582986

[8] A. Misra , “Ethnic‐Specific Criteria for Classification of Body Mass Index: A Perspective for Asian Indians and American Diabetes Association Position Statement,” Diabetes Technology & Therapeutics 17, no. 9 (2015): 667–671, 10.1089/dia.2015.0007. 25902357 PMC4555479

[9] World Health Organization , The Asia‐Pacific Perspective: Redefining Obesity and Its Treatment (World Health Organization, 2000), https://apps.who.int/iris/handle/10665/206936.

[10] World Obesity Federation , The Economic Impact of Overweight & Obesity in 2020 and 2060 (World Obesity Federation, 2025), https://s3‐eu‐west‐1.amazonaws.com/wof‐files/The_Economic_Impact_of_Overweight__Obesity_in_2020_and_2060.pdf.

[11] S. Wharton , D. C. W. Lau , M. Vallis , et al., “Obesity in Adults: A Clinical Practice Guideline,” CMAJ 192, no. 31 (2020): E875–E891, 10.1503/cmaj.191707. 32753461 PMC7828878

[12] M. I. Ullah and S. Tamanna , “Obesity: Clinical Impact, Pathophysiology, Complications, and Modern Innovations in Therapeutic Strategies,” Medicines (Basel, Switzerland) 12, no. 3 (2025): 19. 40843857 10.3390/medicines12030019 PMC12372088

[13] R. M. Anjana , R. Unnikrishnan , M. Deepa , et al., “Metabolic Non‐Communicable Disease Health Report of India: The ICMR‐INDIAB National Cross‐Sectional Study (ICMR‐INDIAB‐17),” Lancet Diabetes and Endocrinology 11, no. 7 (2023): 474–489, 10.1016/s2213-8587(23)00119-5. 37301218

[14] N. H. Phelps , R. K. Singleton , B. Zhou , et al., “Worldwide Trends in Underweight and Obesity From 1990 to 2022: A Pooled Analysis of 3663 Population‐Representative Studies With 222 Million Children, Adolescents, and Adults,” Lancet 403, no. 10431 (2024): 1027–1050, 10.1016/S0140-6736(23)02750-2. 38432237 PMC7615769

[15] I. Mohan , R. Gupta , A. Misra , et al., “Disparities in Prevalence of Cardiometablic Risk Factors in Rural, Urban‐Poor, and Urban‐Middle Class Women in India,” PLoS One 11, no. 2 (2016): e0149437, 10.1371/journal.pone.0149437. 26881429 PMC4755555

[16] NCD Risk Factor Collaboration , Underweight and Obesity in India, 2022 (NCD Risk Factor Collaboration, 2025), https://www.ncdrisc.org/downloads/country‐pdf/double‐burden/NCD‐RisC%20country%20factsheet%20India.pdf.

[17] M. Ng , E. Gakidou , J. Lo , et al., “Global, Regional, and National Prevalence of Adult Overweight and Obesity, 1990–2021, With Forecasts to 2050: A Forecasting Study for the Global Burden of Disease Study 2021,” Lancet 405, no. 10481 (2025): 813–838, 10.1016/S0140-6736(25)00355-1. 40049186 PMC11920007

[18] World Obesity Federation , World Obesity Atlas 2024 (World Obesity Federation, 2025), https://data.worldobesity.org/publications/WOF‐Obesity‐Atlas‐v7.pdf.

[19] National Family Health Survey (NFHS‐5) , 2019–21 India: Volume I (IIPS, 2025), https://dhsprogram.com/pubs/pdf/FR375/FR375.pdf.

[20] V. Khadilkar , N. Shah , R. Harish , et al., “Indian Academy of Pediatrics Revised Guidelines on Evaluation, Prevention and Management of Childhood Obesity,” Indian Pediatrics 60, no. 12 (2023): 1013–1031. 38087786

[21] R. Pradeepa , R. M. Anjana , S. R. Joshi , et al., “Prevalence of Generalized & Abdominal Obesity in Urban & Rural India: The ICMR‐INDIAB Study (Phase‐I) [ICMR‐ NDIAB‐3],” Indian Journal of Medical Research 142, no. 2 (2015): 139–150, 10.4103/0971-5916.164234. 26354211 PMC4613435

[22] A. Misra , N. K. Vikram , A. Ghosh , P. Ranjan , and S. Gulati , “Revised Definition of Obesity in Asian Indians Living in India,” Diabetes & Metabolic Syndrome 19, no. 1 (2025): 102989, 10.1016/j.dsx.2024.102989. 39814628

[23] N. Kapoor , “Thin Fat Obesity: The Tropical Phenotype of Obesity,” in Endotext, ed. K. R. Feingold , B. Anawalt , M. R. Blackman , et al. (MDText.com, Inc., 2000). 33734655

[24] N. Kapoor , M. Lotfaliany , T. Sathish , et al., “Prevalence of Normal Weight Obesity and Its Associated Cardio‐Metabolic Risk Factors ‐ Results From the Baseline Data of the Kerala Diabetes Prevention Program (KDPP),” PLoS One 15, no. 8 (2020): e0237974, 10.1371/journal.pone.0237974. 32841271 PMC7446975

[25] S. Pati , W. Irfan , A. Jameel , S. Ahmed , and R. K. Shahid , “Obesity and Cancer: A Current Overview of Epidemiology, Pathogenesis, Outcomes, and Management,” Cancers (Basel) 15, no. 2 (2023): 485, 10.3390/cancers15020485. 36672434 PMC9857053

[26] S. V. Madhu , K. Nitin , D. Sambit , R. Nishant , and K. Sanjay , “ESI Clinical Practice Guidelines for the Evaluation and Management of Obesity in India,” Indian Journal of Endocrinology and Metabolism 26, no. 4 (2022): 295–318, 10.4103/2230-8210.356236. 36185955 PMC9519829

[27] Global Obesity Observatory , Economic Impact of Overweight and Obesity. Country Results: India (Global Obesity Observatory, 2025), https://data.worldobesity.org/economic‐impact‐new/countries/#IN.

[28] J. J. Jacob , “Tackling the Rising Tide: Understanding the Prevalence of Childhood Obesity in India,” Indian Journal of Endocrinology Metabolism 28, no. 2 (2024): 101–103, 10.4103/ijem.Ijem_144_24. 38911120 PMC11189283

[29] S. Kalra , N. Kapoor , M. Verma , et al., “Defining and Diagnosing Obesity in India: A Call for Advocacy and Action,” Journal of Obesity 2023 (2023): 4178121, 10.1155/2023/4178121. 38026823 PMC10645500

[30] N. R. Deshpande , N. Kapoor , J. J. Dalal , et al., “Consensus on Current Landscape and Treatment Trends of Obesity in India for Primary Care Physicians,” Journal of the Association of Physicians of India 71, no. 10 (2023): 69–77, 10.59556/japi.71.0349. 38716527

[31] A. G. Unnikrishnan , S. Chowdhury , M. M. Garcia , et al., “Perceptions, Attitudes and Barriers to Effective Obesity Care Among People With Obesity and Health Care Professionals in India,” Diabetes, Obesity & Metabolism 26, no. 10 (2024): 4791–4794, 10.1111/dom.15824. 39101238

[32] M. Riaz and S. Lodhi , “Beyond BMI: Exploring Obesity Trends in the South Asian Region,” Obesity Pillars 13 (2025): 100156, 10.1016/j.obpill.2024.100156. 39810860 PMC11732094

[33] J. B. Dixon , R. Abdul Ghani , and P. Sbraccia , “Perceptions of Obesity Among Healthcare Professionals and Policy Makers in 2023: Results of the Global OPEN Survey,” Obesity Science and Practice 11, no. 1 (2025): e70033, 10.1002/osp4.70033. 39781548 PMC11707619

[34] V. Mohan , K. Gokulakrishnan , R. Deepa , C. S. Shanthirani , and M. Datta , “Association of Physical Inactivity With Components of Metabolic Syndrome and Coronary Artery Disease: The Chennai Urban Population Study (CUPS No. 15),” Diabetic Medicine 22, no. 9 (2005): 1206–1211, 10.1111/j.1464-5491.2005.01616.x. 16108850

[35] R. G. Pradeepa and V. Mohan , “Epidemiology of type 2 diabetes in India,” Indian Journal of Ophthalmology 69, no. 11 (2021): 2932–2938, 10.4103/ijo.IJO_1627_21. 34708726 PMC8725109

[36] V. Mohan , V. Sudha , S. Shobana , R. Gayathri , and K. Krishnaswamy , “Are Unhealthy Diets Contributing to the Rapid Rise of Type 2 Diabetes in India?,” Journal of Nutrition 153, no. 4 (2023): 940–948, 10.1016/j.tjnut.2023.02.028. 36858259

[37] D. Shrestha , S. Kalra , N. Somasundaram , et al., “The Kathmandu Declaration: Obesity in the South Asian Region: An Exigency Statement,” Clinical Epidemiology and Global Health 22 (2023): 101315, 10.1016/j.cegh.2023.101315.

[38] D. Shrestha , A. Pankajakshan , A. Rizwan , et al., “The Emergence of the South‐Asia Obesity Forum: A Need of the Hour,” Asian Journal of Obesity 1, no. 1 (2024): 34–36.

[39] S. Kalra , S. Lodhi , S. Selim , et al., “The Colombo Declaration on Obesity Care (CDOC): Person‐, Public‐, and Planner‐Friendly Obesity Management,” Asian Journal of Obesity 1, no. 1 (2024): 7–9.

[40] Press Information Bureau Delhi , Towards a Fit and Healthy India: Combating Obesity Through Collective Action (Press Information Bureau Delhi, 2025), https://pib.gov.in/PressReleasePage.aspx?PRID=2107179.

[41] Medical Council of India , Competency Based Undergraduate Curriculum for the Indian Medical Graduate (Medical Council of India, 2018).

[42] A. G. Bhasker , A. Prasad , P. P. Raj , et al., “OSSI (Obesity and Metabolic Surgery Society of India) Guidelines for Patient and Procedure Selection for Bariatric and Metabolic Surgery,” Obesity Surgery 30, no. 6 (2020): 2362–2368, 10.1007/s11695-020-04497-1. 32125645

[43] T. Mittal , A. Ahuja , A. Dey , S. Agarwal , and R. Goel , “Bariatric and Metabolic Surgery in India: Where Do We Stand?,” Indian Journal of Surgery 8, no. Suppl 3 (2024): 499–506, 10.1007/s12262-021-03149-7.

[44] N. Nadiger , S. Anantharamu , C. N. Priyanka , A. Vidal‐Puig , and A. Mukhopadhyay , “Unique Attributes of Obesity in India: A Narrative Review,” Obesity Medicine 35 (2022): 100454, 10.1016/j.obmed.2022.100454. 38572212 PMC7615800

[45] J. Yoong , V. Schnecke , W. Aekplakorn , et al., “Population‐Level Impact of Weight Loss on Predicted Healthcare Spending and the Incidence of Obesity‐Related Outcomes in the Asia‐Pacific Region: A Modelling Study,” BMC Glob Public Health 2, no. 1 (2024): 68, 10.1186/s44263-024-00094-x. 39681944 PMC11622925

[46] United Nations , Sustainable Development Goals (United Nations, 2025), https://sdgs.un.org/goals.

[47] United Nations , What Are Healthy Diets? Joint Statement by the Food and Agriculture Organization of the United Nations and the World Health Organization (United Nations, 2025), https://iris.who.int/bitstream/handle/10665/379324/9789240101876‐eng.pdf?sequence=5.

[48] W. T. Garvey , J. I. Mechanick , E. M. Brett , et al., “American Association of Clinical Endocrinologists and American College of Endocrinology Comprehensive Clinical Practice Guidelines for Medical Care of Patients With Obesity,” Endocrine Practice 22, no. Suppl 3 (2016): 1–203, 10.4158/ep161365.Gl. 27219496

[49] R. F. Kushner and D. H. Ryan , “Assessment and Lifestyle Management of Patients With Obesity: Clinical Recommendations From Systematic Reviews,” Journal of the American Medical Association 312, no. 9 (2014): 943–952, 10.1001/jama.2014.10432. 25182103

