# Supplementary material for: Burden of Obesity in India: Need for Policy Changes to Attain Highest Possible Level of Health and Well‐Being
Source: Clin Obes. 2026 Feb 2;16(2):e70072. doi: 10.1111/cob.70072 (PMC12865255; doi:10.1111/cob.70072)
Supplement: Supplementary file 1 — Data S1: Supporting Information. [file COB-16-e70072-s001.pdf]

# Burden of obesity in India: Need for policy changes to attain highest possible level of health and well-being

Supporting information

Sanjay Kalra[1], Jothydev Kesavadev[2], Ramen Goel[3], Muffazal Lakdawala[4], Vinayak Agrawal[5], Neena Malhotra[6], Nitin Kapoor[7], Neeta Deshpande[8], Ankush Desai[9], Viswanathan Mohan[10], Rajesh Khadgawat[11], Balram Sharma[12]

[1]Department of Endocrinology, Bharti Hospital, Karnal, India

[2]Jothydev's Diabetes Research Centre, Trivandrum, Kerala, India

[3]Center of Bariatric & Diabetes Surgery, Wockhardt Hospitals, Mumbai, India

[4] Sir H. N. Reliance Foundation Hospital, Mumbai, India

[5]Fortis Memorial Research Institute, Gurugram, India

[6]All India Institute of Medical Sciences, New Delhi, India

[7]Christian Medical College, Vellore, Tamil Nadu, India

[8]Belgaum Diabetes Centre and CentraCare Institute of Diabetes, Obesity and Metabolic Health, Belgaum, Karnataka, India

[9]Department of Endocrinology, Goa Medical College, Bambolim, Goa, India

[10]Madras Diabetes Research Foundation (ICMR Collaborating Centre of Excellence) and Dr. Mohan's Diabetes Specialities Centre (IDF Centre of Excellence in Diabetes Care) Chennai, India

[11]Department of Endocrinology and Metabolism, All India Institute of Medical Sciences, New Delhi, India

[12]SMS Medical College, Jaipur, Rajasthan, India

**Corresponding author details:**

Dr Sanjay Kalra

Department of Endocrinology, Bharti Hospital, Karnal, Haryana 132001, India

E-mail: brideknl@gmail.com

Supplementary table 1 – Results from Indian respondents to the Obesity Disease Burden Study survey - Section B (Obesity History and Experience) and Section C (Views towards weight control, weight loss as an objective and preferred weight management methods) only

| **Q16 - Many different factors impact a person's overall health. How important or not a factor do you consider weight to be in determining your overall health?** | |
|---|---|
| | **N=403** |
| **Important** | **321 (79.6%)** |
| One of the most important factors | 171 (42.4%) |
| A fairly important factor, but some others matter even more | 150 (37.2%) |
| **Not important** | **82 (20.4%)** |
| Of minor importance compared to other factors | 78 (19.4%) |
| Not at all important | 4 (1.0%) |

| **Q17 - To what extent do you agree with the following statement. "I have struggled with excess weight in the past or I am currently struggling"** | |
|---|---|
| | **N=403** |
| **Agree** | **313 (77.8%)** |
| Strongly agree | 236 (58.7%) |
| Somewhat agree | 77 (19.1%) |
| **Neither agree nor disagree** | **33 (8.1%)** |
| **Disagree** | **52 (12.8%)** |
| Somewhat disagree | 31 (7.6%) |
| Strongly disagree | 21 (5.2%) |
| **Don't know** | **5 (1.2%)** |

| **Q18 - Approximately how old were you when you first remember struggling with excess weight? Your best estimate will do (those who answered 'strongly agree' or 'somewhat agree' for Q17)** | |
|---|---|
| | **N=313** |
| Under 13 years old | 64 (20.6%) |
| 13–18 years old | 14 (4.4%) |
| 19–25 years old | 46 (14.8%) |
| 26–35 years old | 72 (23.0%) |
| 36–45 years old | 105 (33.5%) |
| 46–55 years old | 8 (2.5%) |
| 56+ years old | 5 (1.6%) |
| Mean years spent struggling with weight | 10.2 |

| **Q19 - Have you ever been diagnosed with obesity by a medical doctor or qualified healthcare professional?** | |
|---|---|
| | **N=403** |
| Yes | 261 (64.9%) |
| No | 141 (35.1%) |

| **Q20 - Approximately how old were you when you were diagnosed with obesity? Your best estimate will do. (those who answered 'yes' for Q19)** | |
|---|---|
| | **N=261** |
| Under 13 years old | 56 (21.6%) |
| 13–18 years old | 15 (5.7%) |
| 19–25 years old | 31 (11.8%) |
| 26–35 years old | 58 (22.3%) |
| 36–45 years old | 92 (35.3%) |
| 46–55 years old | 8 (3.0%) |
| 56+ years old | 1 (0.4%) |
| Mean age (years) | 27.2 |

| **Q21 - Have you spoken to a healthcare provider about your weight within the past 5 years?** | |
|---|---|
| | **N=403** |
| Yes | 264 (65.6%) |
| No | 138 (34.4%) |

| **Q22 - Which of the following healthcare providers have you talked to about weight loss within the past five years? (those who answered 'yes' for Q21)** | |
|---|---|
| | **N=264** |
| A primary care physician | 145 (55.1%) |
| Dietitian | 145 (55.0%) |
| Obesity specialist | 106 (40.1%) |
| Psychologist or Psychiatrist | 102 (38.6%) |
| Endocrinologist | 101 (38.2%) |
| Another healthcare professional (a nurse, a physician who specializes in a certain condition such as diabetes) | 12 (4.5%) |

| **Q23 - Have you ever made a made a plan to lose weight in the past? By this, we mean taking a decision to try losing weight over a period of time using diet, exercise, medical treatment, lifestyle changes or other approaches. This is regardless of whether you actually lost weight.** | |
|---|---|
| | **N=403** |
| Yes | 338 (84.0%) |
| No | 55 (13.8%) |
| Don’t know/can’t remember | 9 (2.2%) |

| **Q24 - When you made a plan to lose weight in the past, did you try any of the following actions in an effort to achieve weight loss? If you have made such a plan on more than one occasion, please answer based on the last time you did so. Please tick any that apply. (those who answered ‘yes’ for Q23)** | |
|---|---|
| | **N=338** |
| **Diet** | **223 (66.1%)** |
| Generally improving eating habits / reducing calorie intake | 171 (50.5%) |
| Adopting a specific diet or diet program | 140 (41.5%) |
| **Exercise** | **220 (65.2%)** |
| Generally being more active / increasing physical activity | 150 (44.4%) |
| Starting a specific exercise program / joining a gym / getting a personal trainer | 149 (44.1%) |
| **Medical** | **159 (47.2%)** |
| Taking over-the-counter (non-prescription) weight loss medications | 81 (24.0%) |
| Taking prescription weight loss medications | 79 (23.5%) |
| Having behavioural therapy or psychotherapy (e.g., counselling) | 84 (24.9%) |
| Having weight loss surgery / bariatric surgery | 64 (19.1%) |
| **Lifestyle** | **170 (50.3%)** |
| Reducing stress | 115 (34.0%) |
| Improving sleep quality | 121 (35.9%) |

| **Q25 - Which of the following best describes the outcome of the last time you made a plan to lose weight? (those who answered ‘yes’ for Q23)** | |
|---|---|
| | **N=334** |
| **I put on weight** | **79 (23.7%)** |
| **My weight stayed the same** | **70 (21.0%)** |
| **I lost some weight, but have later put it all back on** | **95 (28.4%)** |
| **All who sustained weight loss** | **90 (26.9%)** |
| I lost some weight, but have later put some of it back on | 59 (17.8%) |

| | |
|---|---|
| I lost some weight, and have not since put any back on | 31 (9.1%) |
| **Don’t know/can’t remember (excluded from total N)** | **4** |
| **Q27 - Which one of the following best describes you today?** | |
| | **N=403** |
| I have no (further) plans to lose weight | 86 (21.4%) |
| I sometimes think about losing (further) weight, but it is not high in my priorities | 122 (30.3%) |
| I am not currently taking actions to lose (further) weight, but feel motivated to do so | 78 (19.4%) |
| I am currently taking actions to lose weight | 116 (28.9%) |
| **Q28 - Please indicate whether you are currently trying the following actions in an effort to achieve weight loss. (those who answered ‘I am currently taking actions to lose weight’ for Q27)** | |
| | **N=116** |
| **Diet** | **89 (76.3%)** |
| Generally improving eating habits / reducing calorie intake | 73 (62.8%) |
| Adopting a specific diet or diet program | 60 (51.7%) |
| **Exercise** | **94 (80.6%)** |
| Generally being more active / increasing physical activity | 79 (67.8%) |
| Starting a specific exercise program / joining a gym / getting a personal trainer | 68 (58.6%) |
| **Medical** | **59 (50.9%)** |
| Taking over-the-counter (non-prescription) weight loss medications | 33 (28.0%) |
| Taking prescription weight loss medications | 37 (31.5%) |
| Having behavioural therapy or psychotherapy (e.g., counselling) | 31 (26.3%) |
| Having weight loss surgery / bariatric surgery | 25 (21.3%) |
| **Lifestyle** | **66 (56.8%)** |
| Reducing stress | 53 (45.8%) |
| Improving sleep quality | 52 (45.0%) |
| **Q29 - Please indicate whether you would consider trying the following actions in the future in an effort to achieve weight loss. (those who answered ‘I sometimes think about losing (further) weight, but it is not high in my priorities’ or ‘I am not currently taking actions to lose (further) weight, but feel motivated to do so’ for Q27)** | |
| | **N=200** |
| **Diet** | **128 (63.8%)** |
| Generally improving eating habits / reducing calorie intake | 100 (49.9%) |

| | |
|---|---|
| Adopting a specific diet or diet program | 68 (34.1%) |
| **Exercise** | **130 (64.8%)** |
| Generally being more active / increasing physical activity | 86 (43.0%) |
| Starting a specific exercise program / joining a gym / getting a personal trainer | 75 (37.6%) |
| **Medical** | **112 (56.0%)** |
| Taking over-the-counter (non-prescription) weight loss medications | 57 (28.3%) |
| Taking prescription weight loss medications | 43 (21.3%) |
| Having behavioural therapy or psychotherapy (e.g., counselling) | 40 (20.2%) |
| Having weight loss surgery / bariatric surgery | 29 (14.4%) |
| **Lifestyle** | **112 (55.9%)** |
| Reducing stress | 78 (39.1%) |
| Improving sleep quality | 80 (40.0%) |

**Q30 - Which of the following, if any, have motivated you the most to make a plan to lose weight, today or in the past? Please select all that apply. (those who answered 'Yes; for Q23 and 'I am currently taking actions to lose weight' for Q27)**

| | **N=116** |
|---|---|
| **Health** | **93 (79.7%)** |
| Having general health concerns | 51 (44.1%) |
| Wanting to stop or not need to take medication for a weight-related health condition | 22 (18.8%) |
| Reaching the upper end of the weight range I am comfortable with | 32 (27.2%) |
| Having a specific personal medical event (heart attack, stroke, etc.) or diagnosis (diabetes, liver disease, sleep apnea, etc.) | 28 (23.8%) |
| Wanting to be more fit/in better shape | 62 (53.4%) |
| Wanting to feel better physically, have more energy or be more active | 69 (59.4%) |
| Wanting to improve mental health and wellbeing | 58 (50.0%) |
| **Appearance** | **63 (54.3%)** |
| Wanting to fit into a smaller clothing size | 38 (33.1%) |
| Wanting to be a positive role model for my family/children | 46 (39.9%) |
| **Social** | **85 (72.9%)** |
| An upcoming special occasion or event | 24 (20.5%) |
| Wanting to feel more confident when I am around people | 51 (44.1%) |
| Wanting to improve how I feel about myself | 56 (48.3%) |
| Wanting to improve my dating, relationships and sex life | 36 (31.4%) |

| | |
|---|---|
| Wanting to improve my job performance (e.g., if your job is fairly active) | 38 (33.1%) |
| Because of discriminatory behaviors and comments about my weight made to me in my work environment | 34 (28.9%) |
| Because of discriminatory behaviors and comments about my weight made to me by friends and family | 34 (29.7%) |
| **Life events** | **39 (33.2%)** |
| A major life change such as retirement, divorce, break-up, starting a family | 25 (21.3%) |
| A specific medical event (heart attack, stroke, etc.) or diagnosis (diabetes, liver disease, sleep apnea, etc.) in a family member/close friend | 32 (27.2%) |
| **None of the above/I have no desire to lose weight** | **2 (1.7%)** |

**Q30 - Beyond what you have tried in the past or are trying now, how effective do you believe each of the following actions to be for achieving weight loss in general? Even if you are not sure, we are interested in your perceptions – these could be based on anything: from personal experience, to what you have seen, heard or read elsewhere.**

| | **N=403** |
|---|---|
| **Reducing stress** | |
| Effective | 308 (76.4%) |
| Highly effective | 152 (37.8%) |
| Fairly effective | 155 (38.6%) |
| Slightly effective | 72 (17.9%) |
| Not at all effective | 13 (3.2%) |
| Don’t know | 10 (2.5%) |
| **Improving sleep quality** | |
| Effective | 333 (82.8%) |
| Highly effective | 175 (43.5%) |
| Fairly effective | 158 (39.3%) |
| Slightly effective | 57 (14.2%) |
| Not at all effective | 7 (1.7%) |
| Don’t know | 5 (1.2%) |
| **Taking over-the-counter (non-prescription) weight loss medications** | |
| Effective | 233 (58.0%) |
| Highly effective | 101 (25.1%) |
| Fairly effective | 133 (32.9%) |
| Slightly effective | 76 (18.9%) |
| Not at all effective | 44 (11.0%) |

| | |
|---|---|
| Don’t know | 48 (12.0%) |
| **Taking prescription weight loss medications** | |
| Effective | 236 (58.5%) |
| Highly effective | 112 (27.8%) |
| Fairly effective | 124 (30.7%) |
| Slightly effective | 83 (20.6%) |
| Not at all effective | 35 (8.8%) |
| Don’t know | 49 (12.1%) |
| **Having behavioural therapy or psychotherapy (e.g. counselling)** | |
| Effective | 245 (61.0%) |
| Highly effective | 109 (27.0%) |
| Fairly effective | 137 (34.0%) |
| Slightly effective | 90 (22.4%) |
| Not at all effective | 28 (6.9%) |
| Don’t know | 39 (9.8%) |
| **Having weight loss surgery / bariatric surgery** | |
| Effective | 223 (55.4%) |
| Highly effective | 116 (28.8%) |
| Fairly effective | 107 (26.6%) |
| Slightly effective | 61 (15.2%) |
| Not at all effective | 42 (10.5%) |
| Don’t know | 76 (18.9%) |
| **Generally being more active / increasing physical activity** | |
| Effective | 350 (87.0%) |
| Highly effective | 204 (50.6%) |
| Fairly effective | 146 (36.3%) |
| Slightly effective | 45 (11.1%) |
| Not at all effective | 6 (1.5%) |
| Don’t know | 2 (0.5%) |
| **Starting a specific exercise program / joining a gym / getting a personal trainer** | |
| Effective | 318 (79.1%) |

| | |
|---|---|
| Highly effective | 172 (42.8%) |
| Fairly effective | 146 (36.4%) |
| Slightly effective | 61 (15.3%) |
| Not at all effective | 8 (2.0%) |
| Don’t know | 15 (3.7%) |
| **Generally improving eating habits / reducing calorie intake** | |
| Effective | 336 (83.5%) |
| Highly effective | 192 (47.6%) |
| Fairly effective | 144 (35.9%) |
| Slightly effective | 50 (12.5%) |
| Not at all effective | 9 (2.2%) |
| Don’t know | 7 (1.7%) |
| **Adopting a specific diet or diet program** | |
| Effective | 312 (77.6%) |
| Highly effective | 150 (37.3%) |
| Fairly effective | 162 (40.3%) |
| Slightly effective | 59 (14.8%) |
| Not at all effective | 14 (3.4%) |
| Don’t know | 17 (4.2%) |

| **Q32 - Please indicate your level of agreement with each of the following statements.** | |
|---|---|
| | **N=403** |
| **I have regular access to exercise facilities (at least 1-2 times a week)** | |
| Agree | 310 (76.9%) |
| Strongly agree | 148 (36.7%) |
| Somewhat agree | 162 (40.3%) |
| Neither agree nor disagree | 44 (11.1%) |
| Disagree | 42 (10.6%) |
| Somewhat disagree | 30 (7.4%) |
| Strongly disagree | 13 (3.2%) |
| Don’t know | 6 (1.5%) |
| **There are open spaces that can be used for exercise in my city/residential area** | |

| | |
|---|---|
| Agree | 324 (80.4% |
| Strongly agree | 154 (38.4%) |
| Somewhat agree | 169 (42.0%) |
| Neither agree nor disagree | 40 (9.8%) |
| Disagree | 35 (8.6%) |
| Somewhat disagree | 24 (5.9%) |
| Strongly disagree | 11 (2.7%) |
| Don’t know | 5 (1.2%) |
| **I have access to affordable healthy food options close to where I work** | |
| Agree | 310 (76.9%) |
| Strongly agree | 151 (37.6%) |
| Somewhat agree | 158 (39.3%) |
| Neither agree nor disagree | 49 (12.3%) |
| Disagree | 37 (9.1%) |
| Somewhat disagree | 23 (5.7%) |
| Strongly disagree | 14 (3.4%) |
| Don’t know | 7 (1.7%) |
| **I have access to affordable healthy food options close to my home** | |
| Agree | 318 (78.9%) |
| Strongly agree | 159 (39.6%) |
| Somewhat agree | 158 (39.4%) |
| Neither agree nor disagree | 50 (12.5%) |
| Disagree | 34 (8.3%) |
| Somewhat disagree | 23 (5.6%) |
| Strongly disagree | 11 (2.7%) |
| Don’t know | 1 (0.2%) |
| **I have been discriminated against at work because of my weight** | |
| Agree | 230 (57.0%) |
| Strongly agree | 116 (28.8%) |
| Somewhat agree | 114 (28.2%) |
| Neither agree nor disagree | 51 (12.8%) |
| Disagree | 108 (26.8%) |

| | |
|---|---|
| Somewhat disagree | 35 (8.6%) |
| Strongly disagree | 73 (18.2%) |
| Don’t know | 14 (3.4%) |
| **I have been discriminated against by friends and family because of my weight** | |
| Agree | 241 (60.0%) |
| Strongly agree | 115 (28.6%) |
| Somewhat agree | 126 (31.4%) |
| Neither agree nor disagree | 54 (13.5%) |
| Disagree | 103 (25.5%) |
| Somewhat disagree | 36 (8.8%) |
| Strongly disagree | 67 (16.7%) |
| Don’t know | 4 (1.0%) |
| **Derogatory comments and jokes have been addressed at me because of my weight** | |
| Agree | 256 (63.7%) |
| Strongly agree | 114 (28.3%) |
| Somewhat agree | 142 (35.4%) |
| Neither agree nor disagree | 55 (13.8%) |
| Disagree | 84 (20.9%) |
| Somewhat disagree | 29 (7.3%) |
| Strongly disagree | 55 (13.5%) |
| Don’t know | 7 (1.7%) |
| **People who are overweight are not well accepted in my community** | |
| Agree | 264 (65.6%) |
| Strongly agree | 116 (28.8%) |
| Somewhat agree | 148 (36.9%) |
| Neither agree nor disagree | 59 (14.7%) |
| Disagree | 73 (18.2%) |
| Somewhat disagree | 42 (10.3%) |
| Strongly disagree | 32 (7.9%) |
| Don’t know | 6 (1.5%) |
| **The healthcare system in my country provides adequate support to people who are overweight** | |

| | |
|---|---|
| Agree | 260 (64.7%) |
| Strongly agree | 126 (31.3%) |
| Somewhat agree | 134 (33.4%) |
| Neither agree nor disagree | 52 (13.0%) |
| Disagree | 73 (18.2%) |
| Somewhat disagree | 46 (11.5%) |
| Strongly disagree | 27 (6.6%) |
| Don’t know | 17 (4.2%) |
| **Weight loss success (answered by 369 respondents)** | |
| | **N=369** |
| Successful and maintained | 18 (4.8%) |
| Not successful or maintained | 351 (95.2%) |
| **Mean % weight loss from last attempt** | |
| | **N=403** |
| **Mean % weight loss** | **6.9** |
| ≥5% weight loss | 111 (27.5%) |
| ≥10% weight loss | 53 (13.3%) |
| ≥15% weight loss | 34 (8.4%) |

## Obesity Disease Burden study – Detailed Methodology for the Survey

The online survey in the Obesity Disease Burden study was carried out in adult respondents in Australia, India, South Korea, and Thailand. Using WHO criteria, 400 respondents from India were surveyed (and from each of the other three countries in the study), of which 200 were in the overweight BMI category, and 200 were in the obesity BMI category.

The survey focused on respondents' history with weight issues/obesity, their views towards and experience of weight control, objectives when attempting to lose weight, and their strategies for achieving this.

Respondents were recruited via an existing, general-purpose, web-based consumer panel, with quotas set on age and gender to ensure that the sample responding to the survey was representative of the Indian population. Respondents inputted height and weight measurements to calculate BMI. As such, individuals who have previously been living with overweight or obesity and are now under those thresholds and/or successfully sustained more than 15% loss for a year would not have been included.

Respondents were also excluded if: they declined to disclose their income, they were pregnant, they participate in strength training or bodybuilding programmes and consider themselves to be extremely fit, or if they have had significant weight loss due to major injury or illness in the past 6 months.

The survey was available in local languages, although some Indian respondents would have been excluded as the languages used were Hindi, Tamil, and English. A skew towards urban-based respondents was anticipated and subsequently observed.

# Obesity Disease Burden study – Online Questionnaire

## A. Screener & Respondent Profiling

*Q1) What is your current age?*

*Q2) Which gender do you identify with?*

1. Female
2. Male
3. Other

*Q3) Where do you live in?*

| Australia | South Korea | Thailand | India |
|---|---|---|---|
| **What state do you live in?** | **What province or metropolitan city do you live in?** | **In which region is your primary residence located?** | **In which state or territory is your primary residence located?** |
| 1. Australian Capital Territory<br>2. Christmas Island<br>3. Cocos (Keeling) Islands<br>4. Heard Island and McDonald Islands<br>5. New South Wales<br>6. Northern Territory<br>7. Queensland<br>8. South Australia<br>9. Tasmania<br>10. Victoria<br>11. Western Australia | 1. Busan<br>2. Daegu<br>3. Daejeon<br>4. Gangwon<br>5. Gwangju<br>6. Gyeonggi<br>7. Incheon<br>8. Jeju<br>9. North Chungcheong<br>10. North Gyeongsang<br>11. North Jeolla<br>12. Sejong<br>13. Seoul<br>14. South Chungcheong<br>15. South Gyeongsang<br>16. South Jeolla<br>17. Ulsan | 1. Bangkok<br>2. Central Region (excluding Bangkok)<br>3. Northern Region<br>4. Northeastern Region<br>5. Southern Region | **States:**<br>1. Andhra Pradesh<br>2. Arunachal Pradesh<br>3. Assam<br>4. Bihar<br>5. Chhattisgarh<br>6. Goa<br>7. Gujarat<br>8. Haryana<br>9. Himachal Pradesh<br>10. Jharkhand<br>11. Karnataka<br>12. Kerala<br>13. Madhya Pradesh<br>14. Maharashtra<br>15. Manipur<br>16. Meghalaya<br>17. Mizoram<br>18. Nagaland<br>19. Odisha<br>20. Punjab<br>21. Rajasthan<br>22. Sikkim<br>23. Tamil Nadu<br>24. Telangana<br>25. Tripura<br>26. Uttar Pradesh<br>27. Uttarakhand<br>28. West Bengal<br><br>**Union Territories:**<br>29. Andaman and Nicobar Islands<br>30. Chandigarh<br>31. Dadra & Nagar Haveli and Daman & Diu<br>32. Delhi<br>33. Jammu and Kashmir<br>34. Lakshadweep<br>35. Puducherry<br>36. Ladakh |

*Q4) Which of the following best describes the area you live in?*

1. Urban – Densely populated, city or large town
2. Suburban – Mainly residential, bordering a city or large town
3. Rural – Sparsely populated, small town or village

*Q5) In 2021, what was your income?*

| Australia | South Korea | Thailand | India |
|---|---|---|---|
| **In 2021, how much total combined annual income did all members of your household earn before taxes?** | **In 2021, how much total combined annual income did all members of your household earn before taxes?** | **In 2021, what was your household's total monthly income before taxes?** | **In 2021, what was your household's total yearly income before taxes?** |
| 1. Less than A$15,000<br>2. A$15,000 to A$19,999<br>3. A$20,000 to A$24,999<br>4. A$25,000 to A$29,999<br>5. A$30,000 to A$34,999<br>6. A$35,000 to A$39,999<br>7. A$40,000 to A$44,999<br>8. A$45,000 to A$49,999<br>9. A$50,000 to A$59,999<br>10. A$60,000 to A$74,999<br>11. A$75,000 to A$84,999<br>12. A$85,000 to A$99,999<br>13. A$100,000 to A$124,999<br>14. A$125,000 to A$149,999<br>15. A$150,000 to A$174,999<br>16. A$175,000 to A$199,999<br>17. A$200,000 and above | 1. Less than KRW 15 million<br>2. KRW 15 million to KRW 20 million<br>3. KRW 20 million to KRW 25 million<br>4. KRW 25 million to KRW 30 million<br>5. KRW 30 million to KRW 35 million<br>6. KRW 35 million to KRW 40 million<br>7. KRW 40 million to KRW 45 million<br>8. KRW 45 million to KRW 50 million<br>9. KRW 50 million to KRW 60 million<br>10. KRW 60 million to KRW 70 million<br>11. KRW 70 million to KRW 80 million<br>12. KRW 80 million to KRW 90 million<br>13. KRW 90 million to KRW 100 million<br>14. KRW 100 million to KRW 125 million<br>15. KRW 125 million to KRW 150 million<br>16. KRW 150 million to KRW 200 million<br>17. KRW 200 million or more | 1. <1,500 Baht<br>2. 1,500-3,000 Baht<br>3. 3,001-5,000 Baht<br>4. 5,001-10,000 Baht<br>5. 10,001-15,000 Baht<br>6. 15,001-30,000 Baht<br>7. 30,001-50,000 Baht<br>8. 50,001-100,000 Baht<br>9. 100000+ Baht | 1. Less than 25,000 rupees<br>2. 25,000 to 49,999 rupees<br>3. 50,000 to 99,999 rupees<br>4. 100,000 to 199,999 rupees<br>5. 200,000 to 399,999 rupees<br>6. 400,000 or more rupees |

*Q6) What is the highest level of education you have completed, or the highest degree you have received?*

| Australia | South Korea | Thailand | India |
|---|---|---|---|
| 1. Elementary School > 3rd Grade<br>2. Middle School 4th Grade – 12th Grade<br>3. Completed some high school<br>4. Secondary graduate<br>5. Other post secondary vocational training<br>6. Completed some college<br>7. Associates Degree<br>8. College degree<br>9. Completed some postgraduate<br>10. Masters degree<br>11. Doctorate degree<br>12. None of the above | 1. Some Primary School (Grade 3 or less)<br>2. Primary School (Grades 5-8)<br>3. Secondary School<br>4. Graduated Secondary School<br>5. Vocational Training<br>6. Completed some university, but no degree<br>7. Associate Degree<br>8. College Degree (such as B.A., B.S.)<br>9. Completed some graduate school, but no degree<br>10. Masters degree<br>11. Doctorate degree<br>12. None of the above | 1. Kindergarten<br>2. Primary school<br>3. Secondary school<br>4. High school<br>5. Vocational<br>6. Bachelor's degree<br>7. Post-graduate degree | 1. Secondary school<br>2. Bachelor's degree<br>3. Graduate degree<br>4. Post-graduate<br>5. Doctorate/Ph.D |

*Q7) Are you currently pregnant?*

1. Yes
2. No

*Q8) Do you participate in strength training or bodybuilding programs and consider yourself extremely fit?*

1. Yes
2. No

*Q9) Please enter your height in centimeters (cm). Your best estimate will do.*

*Q10) What is your current weight in kilograms (kg)? Please be as exact as possible.*

*Q11) What is the least you have weighted in the past three years? Your best estimate will do.*

*Q12) What is the most you have weighted in the past three years? Your best estimate will do.*

*Q13) In the past 6 months have you had significant weight loss due to major injury or illness (e.g., cancer, an accident)*

1. Yes
2. No

*Q14) [ONLY FOR THOSE WITH PERCENTAGE WEIGHT LOSS OF 5% OR MORE – SEE Q10 AND Q12] You indicated your current weight is less than your maximum weight within the past three years. For how long would you say you've been able to maintain your weight loss?*

1. 6 months or less 3. A year or more
2. More than 6 months but less than a year 4. I have not been able to maintain weight loss

*Q15) Please indicate for each of the 5 statements which is closest to how you have been feeling over the past 2 weeks.*

| Over the past 2 weeks... | All of the time | Most of the time | More than half the time | Less than half the time | Some of the time | At no time |
|---|---|---|---|---|---|---|
| ...I have felt cheerful and in good spirits | 5 | 4 | 3 | 2 | 1 | 0 |
| ...I have felt calm and relaxed | 5 | 4 | 3 | 2 | 1 | 0 |
| ...I have felt active and vigorous | 5 | 4 | 3 | 2 | 1 | 0 |
| ...I work up feeling fresh and rested | 5 | 4 | 3 | 2 | 1 | 0 |
| ...my daily life has been filled with things that interest me | 5 | 4 | 3 | 2 | 1 | 0 |

**B. Obesity History and Experience**

*Q16) Many different factors impact a person's overall health. How important or not a factor do you consider weight to be in determining your overall health?*

1. Not at all important
2. Of minor importance compared to other factors
3. A fairly important factor, but some others matter even more
4. One of the most important factors

*Q17) To what extent do you agree with the following statement: "I have struggled with excess weight in the past or I'm currently struggling".*

| Don't agree at all | | | | | Strongly agree |
|---|---|---|---|---|---|
| 0 | 1 | 2 | 3 | 4 | 5 |

*Q18) [ONLY FOR THOSE WHO ANSWERED 4 OR 5 FOR Q17] Approximately how old were you when you first remember struggling with excess weight or obesity? Your best estimate will do.*

*Q19) Have you ever been diagnosed with obesity by a medical doctor or qualified health care professional?*

1. Yes
2. No

*Q20) Approximately how old were you when you were diagnosed with obesity? Your best estimate will do.*

*Q21) [ONLY FOR THOSE FORMALLY DIAGNOSED WITH OBESITY – SEE Q19] Have you spoken to a healthcare provider about your weight within the past 5 years?*

1. Yes
2. No

*Q22) [ONLY FOR THOSE WHO ANSWERED 1 FOR Q21] Which of the following healthcare providers have you talked to about weight loss within the past five years?*

1. A primary care physician
2. Dietitian
3. Obesity specialist
4. Psychologist or Psychiatrist
5. Another health care professional (a nurse, a physician who specialises in a certain condition such as diabetes)

**C. Views towards weight control, weight loss as an objective and preferred weight management methods**

*Q23) Have you ever made a made a plan to lose weight in the past? By this, we mean taking a decision to try losing weight over a period of time using diet, exercise, medical treatment, lifestyle changes or other approaches. This is regardless of whether you actually lost weight.*

1. Yes
2. No
3. Don't know/can't remember

*Q24) [ONLY FOR THOSE THAT ANSWERED 1 FOR Q23] When you made a plan to lose weight in the past, did you try any of the following actions in an effort to achieve weight loss? If you have made such a plan on more than one occasion, please answer based on the last time you did so. Please tick any that apply.*

Diet

1. Generally improving eating habits / reducing calorie intake
2. Adopting a specific diet or diet program

Exercise

3. Generally being more active / increasing physical activity
4. Starting a specific exercise program / joining a gym / getting a personal trainer

Medical

5. Taking over-the-counter (non-prescription) weight loss medications
6. Taking prescription weight loss medications
7. Having behavioural therapy or psychotherapy (e.g., counselling)
8. Having weight loss surgery / bariatric surgery

Broader lifestyle

9. Reducing stress
10. Improving sleep quality

*Q25) [ONLY FOR THOSE THAT ANSWERED 1 FOR Q23] Which of the following best describes the outcome of the last time when you made a plan to lose weight?*

1. I put on weight
2. My weight stayed the same
3. I lost some weight, but have later put it all back on
4. I lost some weight, but have later put some of it back on
5. I lost some weight, and have not since put any back on
6. Don’t know/can’t remember

*Q26) [ONLY FOR THOSE THAT ANSWERED 4 OR 5 FOR Q25] How much did you weigh before you last set the objective to lose weight? If you can’t remember exactly, please give an estimate.*

*Q27) Which one of the following best describes you today?*

1. I have no plans to lose weight
2. I sometimes think about losing weight, but it is not high in my priorities
3. I am not currently taking actions to lose weight, but feel motivated to do so
4. I am currently taking actions to lose weight

*Q28) [ONLY FOR THOSE THAT ANSWERED 4 FOR Q27] And for each of the same actions, please indicate whether you are currently trying them in an effort to achieve weight loss. Please tick any that apply.*

Diet

1. Generally improving eating habits / reducing calorie intake
2. Adopting a specific diet or diet program

Exercise

3. Generally being more active / increasing physical activity
4. Starting a specific exercise program / joining a gym / getting a personal trainer

Medical

5. Taking over-the-counter (non-prescription) weight loss medications
6. Taking prescription weight loss medications
7. Having behavioural therapy or psychotherapy (e.g., counselling)
8. Having weight loss surgery / bariatric surgery

Broader lifestyle

9. Reducing stress

10. Improving sleep quality

*Q29) [ONLY FOR THOSE THAT ANSWERED 2 OR 3 FOR Q27] And for each of the same actions, please indicate whether you would consider trying them in future in an effort to achieve weight loss.*

Diet

1. Generally improving eating habits / reducing calorie intake
2. Adopting a specific diet or diet program

Exercise

3. Generally being more active / increasing physical activity
4. Starting a specific exercise program / joining a gym / getting a personal trainer

Medical

5. Taking over-the-counter (non-prescription) weight loss medications
6. Taking prescription weight loss medications
7. Having behavioural therapy or psychotherapy (e.g., counselling)
8. Having weight loss surgery / bariatric surgery

Broader lifestyle

9. Reducing stress
10. Improving sleep quality

*Q30) [ONLY FOR THOSE THAT ANSWERED 1 FOR Q23 AND 4 FOR Q27] Which of the following, if any, have motivated you the most to make a plan to lose weight, today or in the past? Please select all that apply.*

Health

1. Having general health concerns
2. Wanting to stop or not need to take medication for a weight-related health condition
3. Reaching the upper end of the weight range I am comfortable with
4. A specific personal medical event (heart attack, stroke, etc.) or diagnosis (diabetes, liver disease, sleep apnea, etc.)
5. Wanting to be more fit/in better shape
6. Wanting to feel better physically, have more energy or be more active
7. Wanting to improve my mental health and wellbeing

Appearance

8. Wanting to fit into a smaller clothing size
9. Wanting to be a positive role model for my family/children

Social

10. An upcoming special occasion or event
11. Wanting to feel more confident when I am around people
12. Wanting to improve how I feel about myself
13. Wanting to improve my dating, relationships and sex life
14. Wanting to improve my job performance (e.g., if your job is fairly active)
15. Because of discriminatory behaviours and comments targeted at me in my work environment about my weight
16. Because of discriminatory behaviours and comments targeted at me by friends and family about my weight

Life Events

17. A major life change such as retirement, divorce, break-up, starting a family
18. A specific medical event (heart attack, stroke, etc.) or diagnosis (diabetes, liver disease, sleep apnea, etc.) in a family member/close friend
19. Other
20. None of the above/I have no desire to lose weight

*Q31) Beyond what you have tried in the past or are trying now, how effective do you believe each of the following actions to be for achieving weight loss in general? Even if you are not sure, we are interested in your perceptions – these could be based on anything: from personal experience, to what you have seen, heard or read elsewhere.*

| | Not at all effective | Slightly effective | Is fairly effective | Is highly effective | Don't know |
|---|---|---|---|---|---|
| Generally improving eating habits / reducing calorie intake | 1 | 2 | 3 | 4 | 5 |
| Adopting a specific diet or diet program | 1 | 2 | 3 | 4 | 5 |
| Generally being more active / increasing physical activity | 1 | 2 | 3 | 4 | 5 |
| Starting a specific exercise program / joining a gym / getting a personal trainer | 1 | 2 | 3 | 4 | 5 |
| Taking over-the-counter (non-prescription) weight loss medications | 1 | 2 | 3 | 4 | 5 |
| Taking prescription weight loss medications | 1 | 2 | 3 | 4 | 5 |
| Having behavioural therapy or psychotherapy (e.g. counselling) | 1 | 2 | 3 | 4 | 5 |
| Having weight loss surgery / bariatric surgery | 1 | 2 | 3 | 4 | 5 |
| Reducing stress | 1 | 2 | 3 | 4 | 5 |
| Improving sleep quality | 1 | 2 | 3 | 4 | 5 |

*Q32) Please indicate your level of agreement with each of the following statements on a scale from 1 to 10, where 1 is strongly disagree and 10 is strongly agree.*

1. I have regular access to exercise facilities (at least 1-2 times a week)
2. There are open spaces that can be used for exercise in my city/residential area
3. I have access to affordable healthy food options close to where I work
4. I have access to affordable healthy food options close to my home
5. I have been discriminated against at work because of my weight
6. I have been discriminated against by friends and family because of my weight
7. Derogatory comments and jokes have been addressed at me because of my weight
8. People with overweight are not well accepted in my community
9. The healthcare system in my country provides adequate support to people with overweight

**D. Impact of COVID-19**

*Q33) Please think back to when your country had the strictest set of measures to combat the Covid-19 pandemic. At this time did you find it easier or harder to do each of the following, compared to before the pandemic?*

| | Much harder | Somewhat harder | About the same | Somewhat easier | Much easier | Not applicable/ Don't know |
|---|---|---|---|---|---|---|
| Exercise regularly (at least 1-2 times a week) | 1 | 2 | 3 | 4 | 5 | 6 |
| Discuss ongoing health issues with a healthcare practitioner | 1 | 2 | 3 | 4 | 5 | 6 |
| Consume a nutritious and varied diet | 1 | 2 | 3 | 4 | 5 | 6 |
| Meet financial obligations (loan repayments, household bills, etc.) | 1 | 2 | 3 | 4 | 5 | 6 |
| Control the amount of alcohol you consume | 1 | 2 | 3 | 4 | 5 | 6 |
| Control the amount of tobacco/tobacco products you consume | 1 | 2 | 3 | 4 | 5 | 6 |
| Do things outside of your home | 1 | 2 | 3 | 4 | 5 | 6 |

*Q34) And how about now – do you find it easier or harder to do each of the following, compared to before the pandemic?*

| | Much harder | Somewhat harder | About the same | Somewhat easier | Much easier | Not applicable/ Don't know |
|---|---|---|---|---|---|---|
| Exercise regularly (at least 1-2 times a week) | 1 | 2 | 3 | 4 | 5 | 6 |
| Discuss ongoing health issues with a healthcare practitioner | 1 | 2 | 3 | 4 | 5 | 6 |
| Consume a nutritious and varied diet | 1 | 2 | 3 | 4 | 5 | 6 |
| Meet financial obligations (loan repayments, household bills, etc.) | 1 | 2 | 3 | 4 | 5 | 6 |
| Control the amount of alcohol you consume | 1 | 2 | 3 | 4 | 5 | 6 |
| Control the amount of tobacco/tobacco products you consume | 1 | 2 | 3 | 4 | 5 | 6 |
| Do things outside of your home | 1 | 2 | 3 | 4 | 5 | 6 |

*Q35) Regardless of whether you have previously tested positive for Covid, do you believe that were you to catch it in future you would…*

1. …Become severely ill
2. …Become moderately ill
3. …Become mildly ill
4. Don't know/can't remember

*Q36) [ONLY FOR THOSE THAT ANSWERED 1 OR 2 FOR Q35] Which one of the following best describes how you feel about the impact of weight on Covid symptoms, were you to catch it in future?*

1. My weight means I'm highly likely to experience severe Covid symptoms
2. My weight means I'm somewhat more likely to experience severe Covid symptoms
3. My weight gives me a slight chance of experiencing severe Covid symptoms
4. My weight has nothing to do with the symptoms of Covid I might experience
5. Don't know/can't remember

*Q37) Would you say you are less likely today compared to before the pandemic to do each of the following due to the prospect of catching Covid-19? Please select any that apply.*

1. Going to the gym or exercising indoors
2. Exercising outdoors
3. Going to shops or markets
4. Going to my place of work
5. Socialising
6. Going to events
7. Don't know/can't remember

*Q38) Compared to before the pandemic, how important are each of the following to you?*

| | Much more important than before | Somewhat more important than before | About the same as before | Somewhat less important than before | Much less important than before | Don't know |
|---|---|---|---|---|---|---|
| Controlling my weight | 1 | 2 | 3 | 4 | 5 | 6 |
| Avoiding transmittable illnesses | 1 | 2 | 3 | 4 | 5 | 6 |
| Living a generally healthy lifestyle | 1 | 2 | 3 | 4 | 5 | 6 |
